# Assessing the colony morphotypes and antibiotic susceptibility profile of Malaysian clinical *Burkholderia pseudomallei* to support the use of EUCAST disk diffusion breakpoints to determine antibiotic resistance

**DOI:** 10.1007/s10096-023-04707-5

**Published:** 2023-11-24

**Authors:** Shirley Yi Fen Hii, Nurul Naziha Zaiful Bahrina, Maswani Nabilah Mohd Zaidi, Rohaidah Hashim, Norazah Ahmad

**Affiliations:** Bacteriology Unit, Infectious Diseases Research Centre, Institute for Medical Research, National Institutes of Health, Ministry of Health Malaysia, Jalan Setia Murni U13/52, Seksyen U13 Setia Alam, 40170 Shah Alam, Selangor Malaysia

**Keywords:** Antibiotic susceptibility testing, *Burkholderia pseudomallei*, Disk diffusion, Broth microdilution, Colony morphotypes

## Abstract

*Burkholderia pseudomallei* is intrinsically resistant to many antibiotics. This study aimed to assess bacterial colony morphotypes and the validity of using disk diffusion method (DD) to determine antibiotic resistance in Malaysian clinical *B. pseudomallei* isolates for ceftazidime (CAZ), meropenem (MEM), amoxicillin-clavulanate (AMC) and doxycycline (DOX). DD produced good categorical agreements exhibiting concordance of 100% with reference method, broth microdilution for CAZ and DOX, 98.6% for MEM and 97.2% for AMC. Smooth-centred colonies were most frequently observed. EUCAST DD interpretative criterion is suitable to interpret *B. pseudomallei* CAZ, MEM, AMC and DOX resistance. Increasing AMC MIC in *B. pseudomallei* is a concern.

**Supplementary Information:**

The online version contains supplementary material available at 10.1007/s10096-023-04707-5.

## Introduction

*Burkholderia pseudomallei* is the causative agent for melioidosis, a potentially fatal infection endemic in Southeast Asia and northern Australia. The current mortality rate remains high at 35–54% [1, 2] and no vaccine available. *B. pseudomallei* is intrinsically resistant to multiple antibiotics. The use of appropriate antibiotic treatment at early stage including initial phase, ceftazidime (CAZ) or meropenem (MEM) or imipenem (IPM), followed by eradication phase, trimethoprim-sulfamethoxazole (SXT), showed reduced mortality rate [3].

Initial in vitro antibiotic susceptibility testing (AST) screening plays an important role to decide appropriate antibiotics administered and avoid therapeutic failure. The gold standard is broth microdilution method (BMD), and there is no interpretative criteria for DD according to the Clinical and Laboratory Standards Institute M45 (CLSI) [4]. Despite that, disk diffusion testing (DD) is preferable for routine AST due to its low cost and being less laborious. Closely related species such as *B. cepacia* or *Pseudomonas aeruginosa* were usually referred to due to absence of standardized interpretative criteria [4, 5]. In this study, we aimed to evaluate the use of recently established EUCAST DD interpretative criteria [6, 7] to determine antibiotic resistance based on inhibition zone diameter breakpoints with reference to CLSI [4] for AST reporting of Malaysian *B. pseudomallei* clinical isolates. In addition, we also examine if the colony morphotypes on Ashdown agar (ASH) affect antibiotic susceptibility.

## Methods

A total of 143 *B. pseudomallei* clinical isolates that included eight additional isolates (different colony morphotypes from same sample) were collected from 135 patients between year 2013 and 2018 from hospitals in Malaysia. The isolates were collected from various clinical samples including blood and pus (Table S1). The isolates were confirmed as *B. pseudomallei* by biochemical tests, PCR and typical appearance on Ashdown agar. All bacterial isolates were grown and tested in biosafety level 3 (BSL3) laboratory. The isolates were tested with both DD and BMD against CAZ, MEM, AMC and doxycycline (DOX). All antibiotic powders were purchased from Sigma, MCE and Santa Cruz Biotechnology and antibiotic disks from Oxoid. The quality control strains used were *Escherichia coli* ATCC25922, *E. coli* ATCC35218 and *P. aeruginosa* ATCC27853. The procedure was performed in triplicate as described [1]. Inhibition zone diameters by DD were compared with MIC values by BMD following interpretative criteria by EUCAST [6] and CLSI M45 [4], respectively, except MEM that utilizes EUCAST MIC breakpoints as there is no interpretative criterion for MEM in CLSI. Categorical comparisons of the AST results were performed using simple correlation and linear regression (SPSS Statistics, Version 23.0). All isolates were cultured on ASH for 4 days at 37 °C. The colony morphotypes for each isolate were recorded and grouped (types 1 to 7) based on criteria as described by Chantratita et al. [8].

## Results

**Fig. 1:**
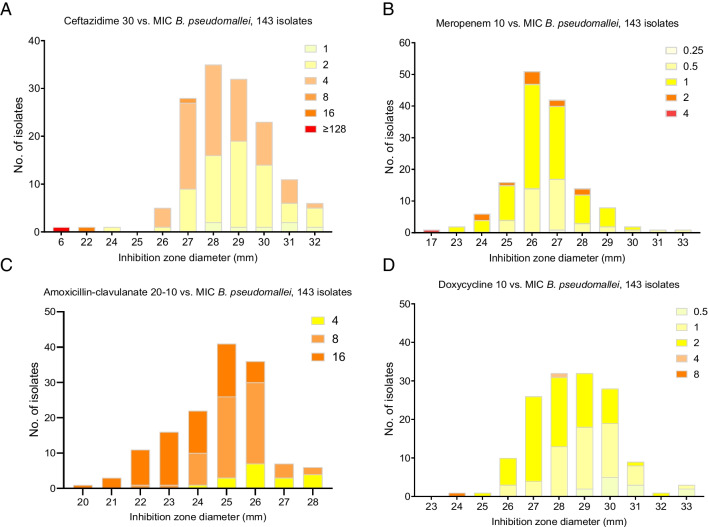
Antibiotic MIC-inhibition zone diameter distributions of *B. pseudomallei* for **A** ceftazidime, **B** meropenem, **C** amoxicillin-clavulanate and **D** doxycycline. Corresponding MIC values are shown through the colouring of bars. Red bar indicates resistant (R) which correspond to CLSI M45 MIC breakpoints for *B. pseudomallei* except meropenem by EUCAST MIC breakpoint

The MIC-inhibition zone diameter distributions for CAZ, MEM, AMC and DOX against *B. pseudomallei* are displayed (Fig. 1). The mode and distributions of DD zone diameters and BMD MIC are 28, 6–32 mm and 4, 1–128 μg/ml (CAZ); 26, 17–32 mm and 1, 0.25–4 μg/ml (MEM); 25, 20–28 mm and 8, 4–16 μg/ml (AMC); and 28 and 29, 24–33 mm and 2, 0.5–8 μg/ml (DOX) (Fig. 1 and Tables 1 and 2). EUCAST recommends the use of susceptible, increased exposure (SI) instead of intermediate-resistant (I) to indicate higher dose antibiotics required for the isolates resides between susceptible (S) and resistant (R) category [7]. In this study, (S), (SI) and (I) were used to indicate non-resistant isolates. DD produced good categorical agreements to differentiate antibiotic-resistant and non-resistant isolates, exhibiting concordance of 100% with BMD for CAZ and DOX, 98.6% for MEM and 97.2% for AMC. Pearson’s correlations between DD and BMD were 0.69 and 0.45 (*p* < 0.01) for CAZ and MEM, respectively. Sixty-two AMC-intermediate-resistant isolates (16 μg/ml) based on CLSI MIC cutoff were termed as non-resistant and within the (SI) range (22–49 mm) except four isolates interpreted as R by DD at 20–21 mm (resistant, < 22 mm). The MIC_50_ was 4 μg/ml, 1 μg/ml, 8 μg/ml and 2 μg/ml and MIC_90_ was 4 μg/ml, 2 μg/ml, 16 μg/ml and 2 μg/ml for CAZ, MEM, AMC and DOX, respectively.

**Table 1:**
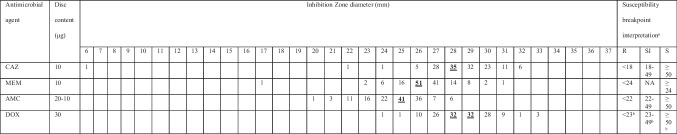
Inhibition zone diameter distributions for *B. pseudomallei* clinical isolates against antibiotics, *n* = 143

^a^Inhibition zone diameter breakpoint interpretation by the European Committee on Antimicrobial Susceptibility Testing (EUCAST)

^b^Tetracycline breakpoint interpretation

Mode inhibition zone diameters are underlined and bolded

**Table 2:** Minimum inhibitory concentration (MIC) distributions for *B. pseudomallei* clinical isolates, *n* = 143

Antimicrobial agent	MIC (μg/ml)										MIC breakpoint interpretation^a^		
	0.25	0.5	1	2	4	8	16	32	64	≥ 128	R	I	S
CAZ			7	64	**69**	1	1			1	≥ 32	16	≤ 8
MEM	1	41	**89**	11	1							NA	
AMC					18	**63**	62				≥ 32/16	16/8	≤ 8/4
DOX		12	56	**73**	1	1					≥ 16	8	≤ 4

^a^MIC interpretation breakpoint by Clinical and Laboratory Standards Institute (CLSI)

*NA*, not applicable

Mode minimum inhibitory concentrations are underlined and bolded

In this study, 19 of 143 *B. pseudomallei* isolates do not grow on ASH. The most frequently observed colony morphotypes were types VI, III and IV each at > 20% with smooth surface in the centre of colony (Table 3). Further investigation on the antibiotic susceptibility profile showed that reduced antibiotic susceptibility for AMC was observed in all colony morphotypes except type V with highest frequency in type VI. CAZ-R, CAZ-I and MEM-R were observed in each of types VII, I and IV, respectively. Among 135 patients’ samples, an additional eight different *B. pseudomallei* colony morphotypes were observed on ASH from eight patients’ samples. The isolates were cultured from IMRS54 (A and B), IMRS71 (A and B), IMRS101 (M and W), IMRS103 (A and B), IMRS131 (P and D), IMRS166 (P and D), IMRS187 (A and B) and IMRS195 (A and B) (Table S1). Among the eight samples, combination of colony morphotypes III-VI were most commonly observed (37.5%) followed by III-IV (25%), II-VI (25%) and IV-VI (12.5%). All showed similar antibiotic susceptibility pattern between both colony morphotypes. The details of each colony morphotype and antibiotic susceptibility are described in Table S1.

**Table 3:**
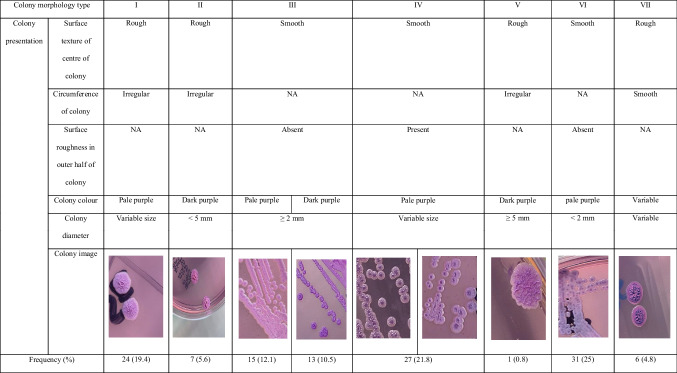
Colony morphotypes of *B. pseudomallei* clinical isolates on Ashdown agar, *n* = 124

*NA*, not applicable

## Discussion

This study compared EUCAST DD interpretative criteria to reference CLSI BMD cutoffs to determine antibiotic resistance for Malaysian clinical *B. pseudomallei* isolates. CLSI is the primary reference for bacterial AST reporting in local hospital setting [9]. There is no CLSI DD breakpoints for *B. pseudomallei*; hence, this data will be beneficial to standardize EUCAST DD method as the more feasible way of testing for melioidosis in local settings. The antibiotic susceptibility profile of different colony morphotypes was also evaluated. In Malaysia, CAZ is the first-line antibiotic for melioidosis treatment and MEM as backup option for severe infections followed by eradication therapy by SXT whilst AMC as alternative for both treatment phases [10]. We observed that the inhibition zone diameter proposed by EUCAST is in concordance with CLSI MIC breakpoints and suitable to be used to interpret AST by DD in our local setting. In comparison to earlier study by Ahmad et al., the mode MIC of CAZ and AMC has increased > twofold from 2 to 4 and 8 μg/ml, respectively. The AMC MIC_90_ has escalated to 16 μg/ml (AMC-I) in this study from 4 μg/ml (AMC-S) [5]. The reducing susceptibility to AMC (AMC-I, 44%) over the years is alarming and requires attention. The improper use of AMC may induce development of antibiotic resistance [11]. A recent survey from several centres (Table S2) [12, 13] showed similar inhibition zone diameter and MIC distributions with results generated from this study for all antibiotics tested except AMC and DOX that showed slightly higher MIC mode in this study. EUCAST employed CAZ disk content of 10 μg instead of 30 μg by CLSI for DD [7]. Despite that, we observed that CAZ breakpoint (resistant, < 18 mm) by EUCAST is able to differentiate wild-type from resistant isolates based on CLSI MIC cutoffs. We also observed that if EUCAST MIC breakpoints were applied for AMC, the AMC-R isolates (> 8 μg/ml) increase to 44% and only 59.4% agrees between DD and BMD. The discrepancy could be due to fixed amount of clavulanate at 2 μg/ml regardless of amoxicillin concentration according to EUCAST BMD whereas AMC were performed in 2:1 ratio of amoxicillin/clavulanate by CLSI BMD [4, 7].

The 19 non-growing *B. pseudomallei* isolates on ASH were from Sarawak in line with previous report on the presence of gentamicin-sensitive isolates in Sarawak. A non-synonymous amino acid change in amrB of the AmrAB-OprA efflux pump is reported to be the cause of the alteration in gentamicin susceptibility [14]. Majority of *B. pseudomallei* in this study (70%) present smooth centre-surface differed with Thailand observing predominantly rough centre-surface colony [8, 12]. Small and slow-growing *B. pseudomallei* colonies have been associated with decreased susceptibility to CAZ, MEM, DOX and trimethoprim-sulfamethoxazole [1, 15]. However, we do not observe the presence of these colonies in this study. The presence of > 1 colony morphotypes in clinical *B. pseudomallei* as observed in this study is not uncommon. Despite low number of samples, we have observed two different colony morphotypes from blood, pus and sputum which is similar to the report of Chantratita et al. [8]. The genetic characteristics of these isolates will warrant a further study. A limitation to this study is the limited information of the patients’ history and varying distributions of colony morphotypes to be associated with antibiotic resistance.

## Conclusions

The outcome of this study supports the use of zone diameter breakpoints proposed by EUCAST as complementary to CLSI MIC cutoffs and applicable for *B. pseudomallei* AST interpretation in local setting where DD is used routinely. Overall, the prevalence of antibiotic resistance against melioidosis first-line antibiotics is low in Malaysian clinical *B. pseudomallei* isolates. Smooth centre-surface is the dominant colony morphotypes in Malaysia.

### Supplementary Information

Below is the link to the electronic supplementary material.

Supplementary file1 (XLSX 31 KB)

Supplementary file2 (TIF 5618 KB)

Supplementary file3 (TIF 2906 KB)

Supplementary file4 (TIF 2266 KB)

Supplementary file5 (TIF 2547 KB)

Supplementary file6 (TIF 2065 KB)

Supplementary file7 (TIF 398 KB)

Supplementary file8 (TIF 2783 KB)

Supplementary file9 (TIF 2361 KB)

Supplementary file10 (TIF 2716 KB)

## Data Availability

The datasets generated during and/or analysed during the current study are available in this manuscript.

## References

[1] Hii SYF, Tandhavanant S, Phunpang R, Ekchariyawat P, Saiprom N, Chewapreecha C (2021). Antibiotic susceptibility of clinical *Burkholderia*
*pseudomallei* isolates in northeast Thailand during 2015–2018 and the genomic characterization of β-lactam-resistant isolates. Antimicrob Agents Chemother.

[2] Mariappan V, Vellasamy KM, Anpalagar RR, Yue-Min L, Zainal Abidin N, Subramaniam S (2022). One Health surveillance approaches for melioidosis and glanders: the Malaysian perspective. Front Vet Sci.

[3] Dance D (2014). Treatment and prophylaxis of melioidosis. Int J Antimicrob Agents.

[4] CLSI (2016) Methods for antimicrobial dilution and disc susceptibility testing of infrequently isolated or fastidious bacteria. 3rd ed. CLSI guideline M45. Wayne, PA: Clinical and Laboratory Standards Institute. Accessed May 17, 2023

[5] Ahmad N, Hashim R, Mohd Noor A (2013) The in vitro antibiotic susceptibility of Malaysian isolates of *Burkholderia pseudomallei*. Int J Microbiol 121845. 10.1155/2013/121845 10.1155/2013/121845 PMC3803121 24194761

[6] Dance DAB, Wuthiekanun V, Baird RW, Norton R, Limmathurotsakul D, Currie BJ (2021). Interpreting *Burkholderia*
*pseudomallei* disc diffusion susceptibility test results by the EUCAST method. Clin Microbiol Infect.

[7] The European Committee on Antimicrobial Susceptibility Testing (EUCAST) (2023) Breakpoint tables for interpretation of MICs and zone diameters. Version 13.0, http://www.eucast.org. Accessed May 17, 2023

[8] Chantratita N, Wuthiekanun V, Boonbumrung K, Tiyawisutsri R, Vesaratchavest M, Limmathurotsakul D (2007). Biological relevance of colony morphology and phenotypic switching by *Burkholderia*
*pseudomallei*. J Bacteriol.

[9] National Antibiotic Resistance Surveillance Report (2022) Institute for Medical Research, National Institutes of Health Malaysia. Available at https://imr.nih.gov.my/images/uploads/NSAR/2022/NSAR-REPORT_2022_to-be-published.pdf.

[10] National Antimicobial Guideline, Ministry of Health Malaysia. 3rd ed. September 2019.

[11] Aslam A, Zin CS, Ab Rahman NS, Gajdács M, Ahmed SI, Jamshed S (2021). Self-medication practices with antibiotics and associated factors among the public of Malaysia: a cross-sectional study. Drug Healthc Patient Saf.

[12] Karatuna O, Dance DAB, Matuschek E, Åhman J, Turner P, Hopkins J (2020). *Burkholderia*
*pseudomallei* multi-centre study to establish EUCAST MIC and zone diameter distributions and epidemiological cut-off values. Clin Microbiol Infect.

[13] Prommachote W, Mala W, Songsri J, Khoosuilee J, Wansu S, Srisara J (2022). Diversity of colony morphotypes, biochemical characteristics, and drug susceptibility patterns of *Burkholderis*
*pseudomallei* isolated from humans, animals, and environmental sources in Thailand. Trends Sci.

[14] Podin Y, Sarovich DS, Price EP, Kaestli M, Mayo M, KingChing H (2014). *Burkholderia*
*pseudomallei* isolates from Sarawak, Malaysian Borneo, are predominantly susceptible to aminoglycosides and macrolides. Antimicrob Agents Chemother.

[15] Wang X, Zheng X, Huang M, Liu L (2020). A comparative genomic analysis of small-colony variant and wild-type *Burkholderia*
*pseudomallei* in a patient with bacterial liver abscess. J Glob Antimicrob Resist.

