# Supplementary figures and images for: Assessing the colony morphotypes and antibiotic susceptibility profile of Malaysian clinical Burkholderia pseudomallei to support the use of EUCAST disk diffusion breakpoints to determine antibiotic resistance
Source: Eur J Clin Microbiol Infect Dis. 2023 Nov 24;43(2):373–8. doi: 10.1007/s10096-023-04707-5 (PMC10822001; doi:10.1007/s10096-023-04707-5)

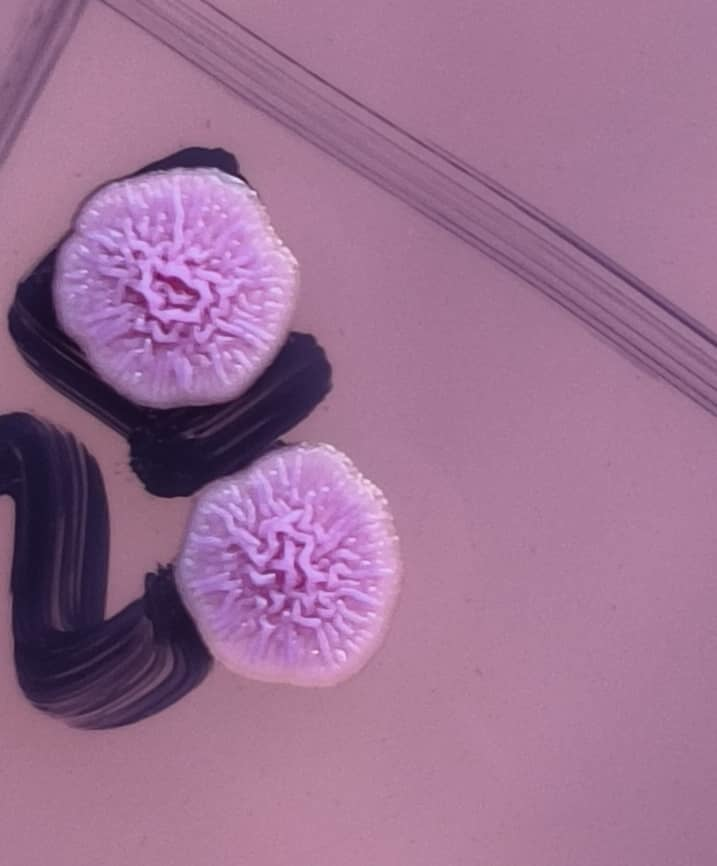

Supplement: Supplementary file 2 — Supplementary file2 (TIF 5618 KB) [file 10096_2023_4707_MOESM2_ESM.tif]

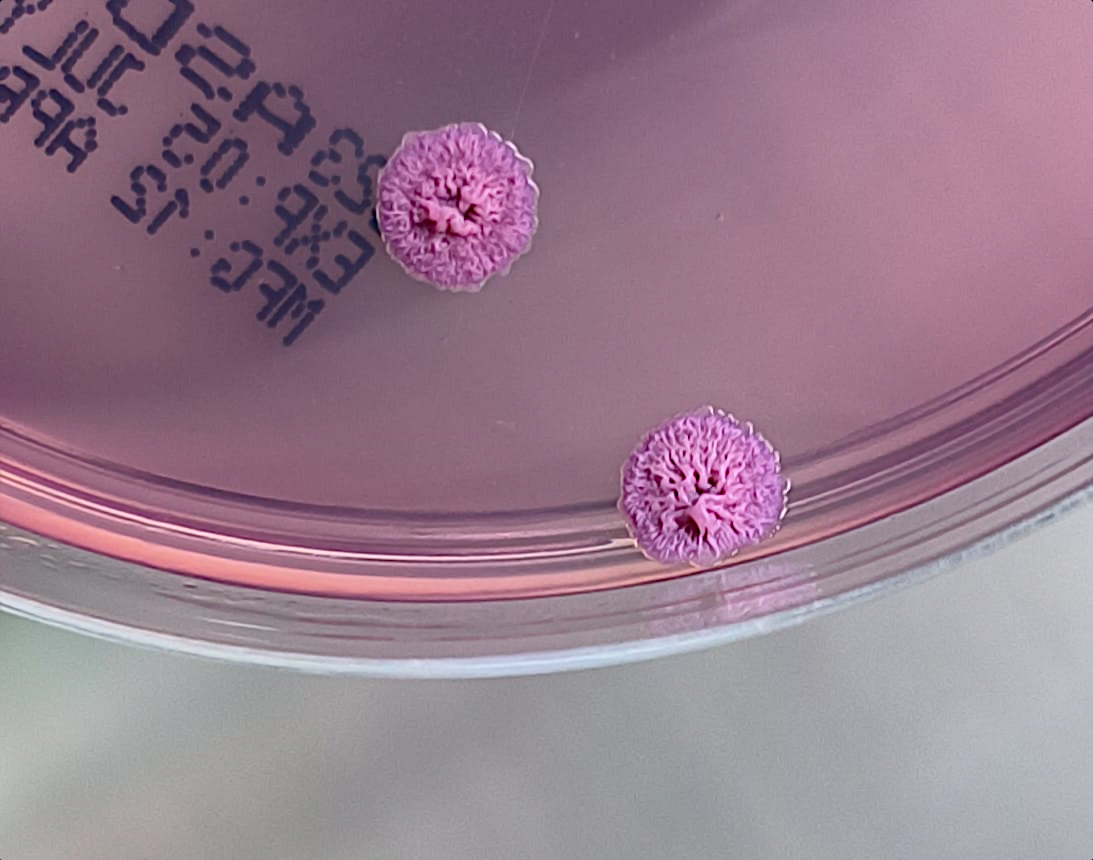

Supplement: Supplementary file 3 — Supplementary file3 (TIF 2906 KB) [file 10096_2023_4707_MOESM3_ESM.tif]

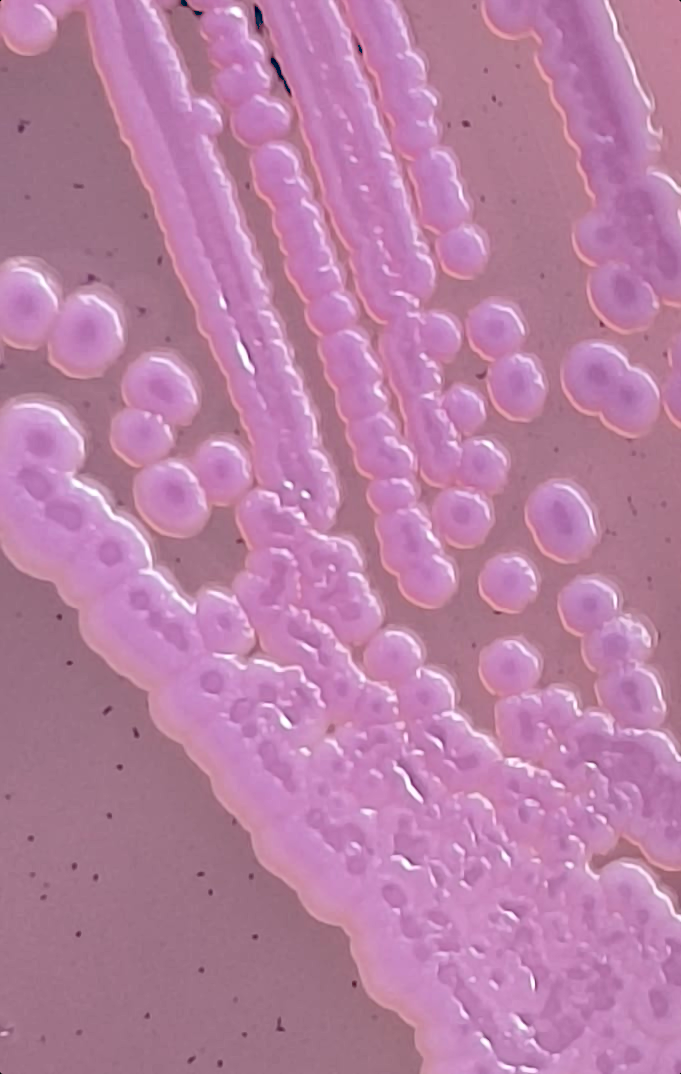

Supplement: Supplementary file 4 — Supplementary file4 (TIF 2266 KB) [file 10096_2023_4707_MOESM4_ESM.tif]

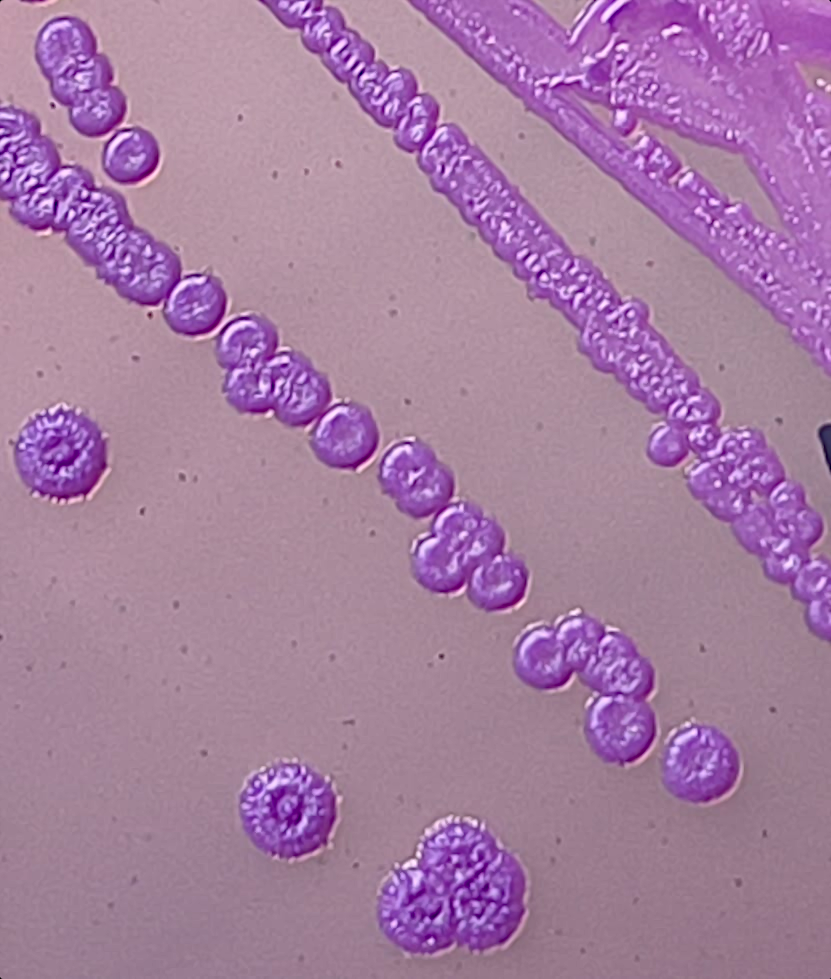

Supplement: Supplementary file 5 — Supplementary file5 (TIF 2547 KB) [file 10096_2023_4707_MOESM5_ESM.tif]

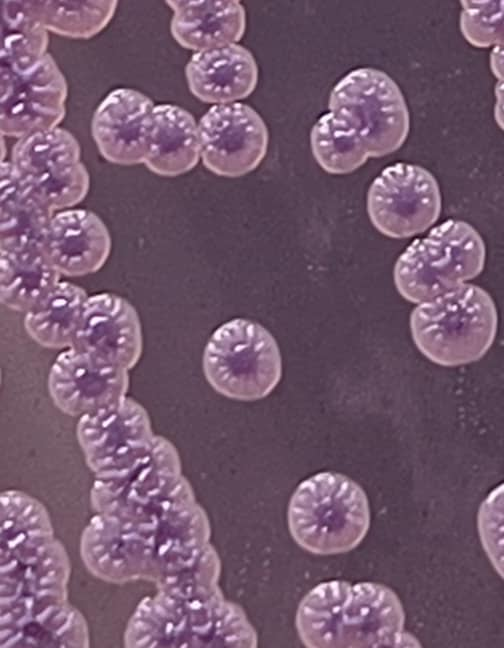

Supplement: Supplementary file 6 — Supplementary file6 (TIF 2065 KB) [file 10096_2023_4707_MOESM6_ESM.tif]

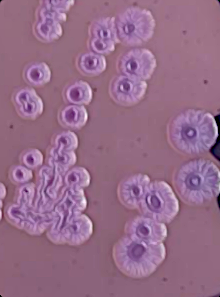

Supplement: Supplementary file 7 — Supplementary file7 (TIF 398 KB) [file 10096_2023_4707_MOESM7_ESM.tif]

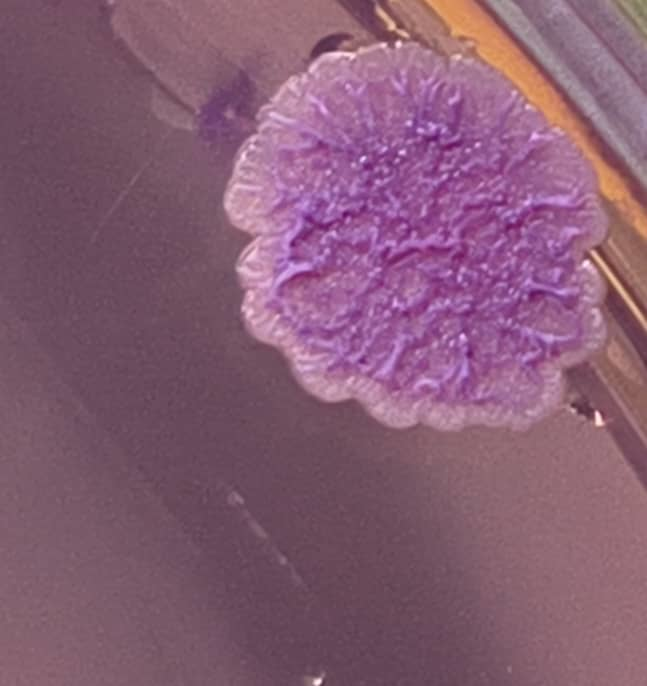

Supplement: Supplementary file 8 — Supplementary file8 (TIF 2783 KB) [file 10096_2023_4707_MOESM8_ESM.tif]

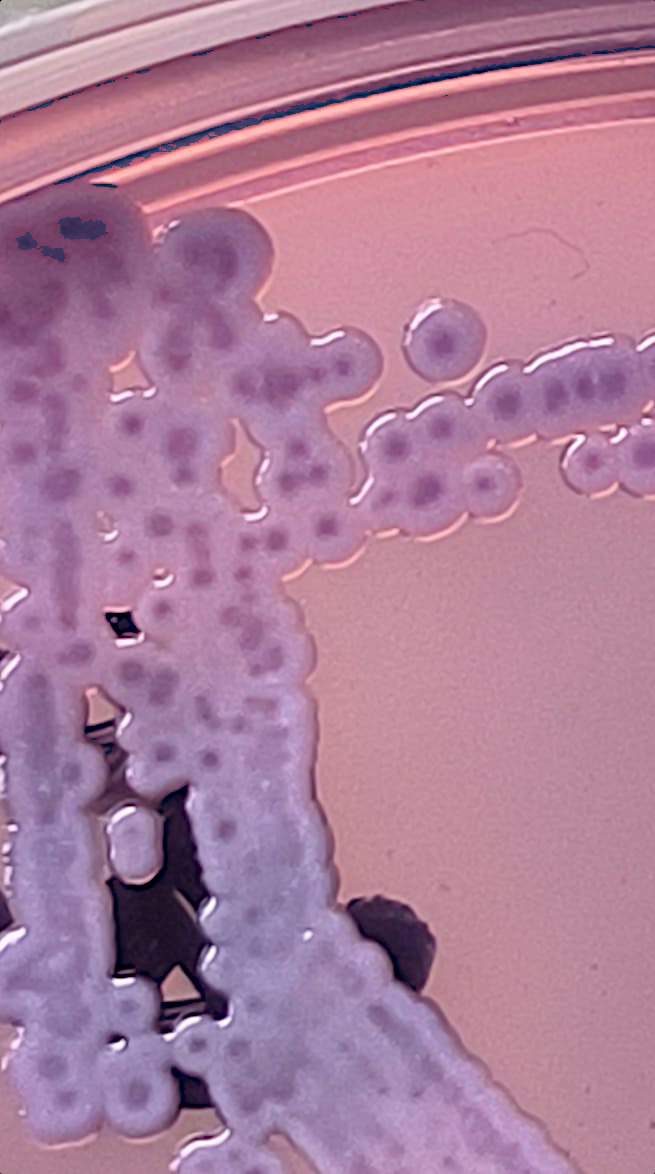

Supplement: Supplementary file 9 — Supplementary file9 (TIF 2361 KB) [file 10096_2023_4707_MOESM9_ESM.tif]

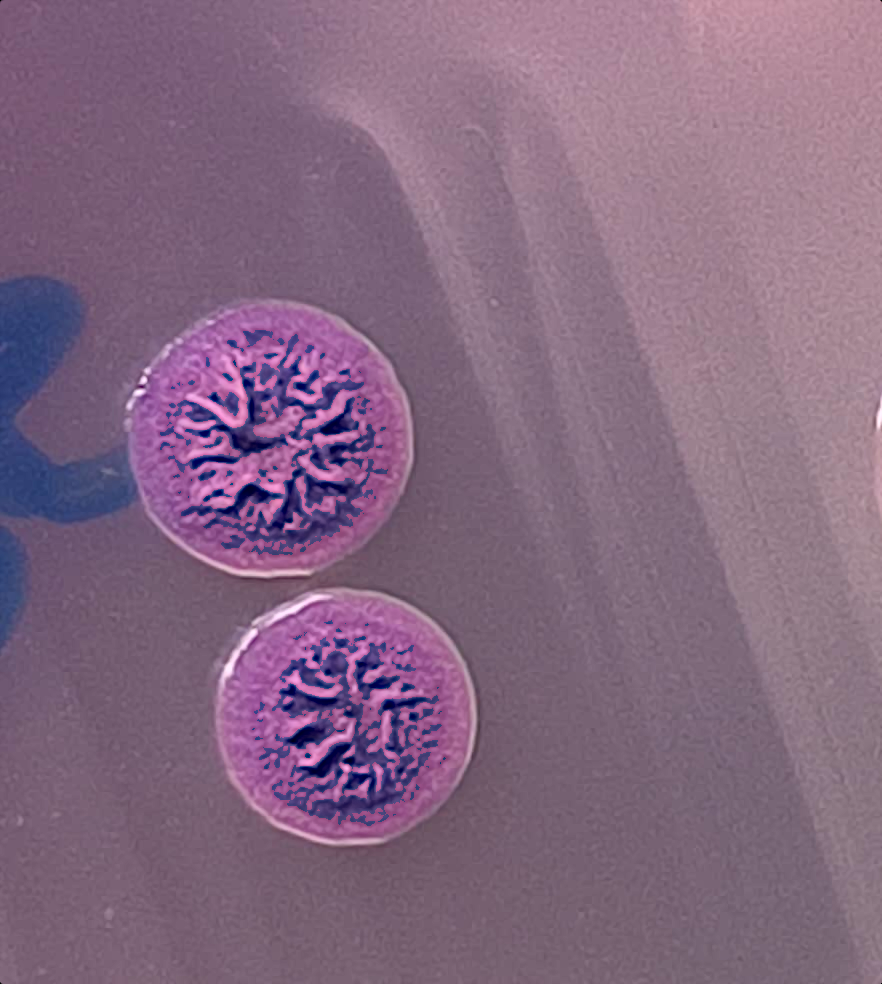

Supplement: Supplementary file 10 — Supplementary file10 (TIF 2716 KB) [file 10096_2023_4707_MOESM10_ESM.tif]
